# Supplementary material for: Promoting photocatalytic CO2 reduction with a molecular copper purpurin chromophore
Source: Nat Commun. 2021 Mar 23;12:1835. doi: 10.1038/s41467-021-21923-9 (PMC7987958; doi:10.1038/s41467-021-21923-9)
Supplement: Supplementary file 1 — Supplementary Information [file 41467_2021_21923_MOESM1_ESM.pdf]

Supporting Information

Promoting photocatalytic CO_2 reduction with a molecular copper purpurin chromophore

Yuan et al.

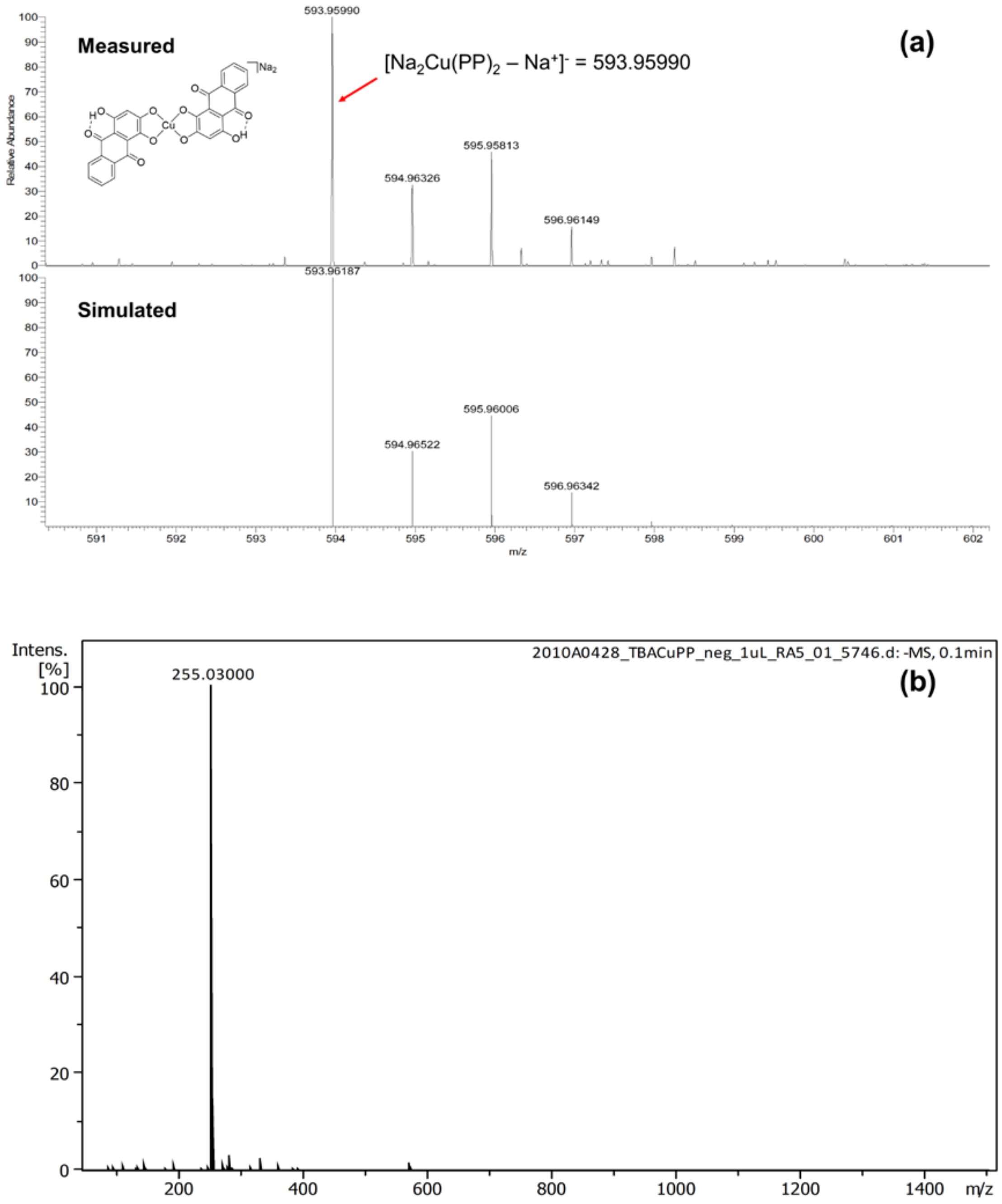

Supplementary Figure 1. HRMS spectra. HRMS spectra of $Na_2Cu(PP)_2$ (a) and $(TBA)_2(CuPP)_2$ (b) in CH_3OH (negative ion mode).

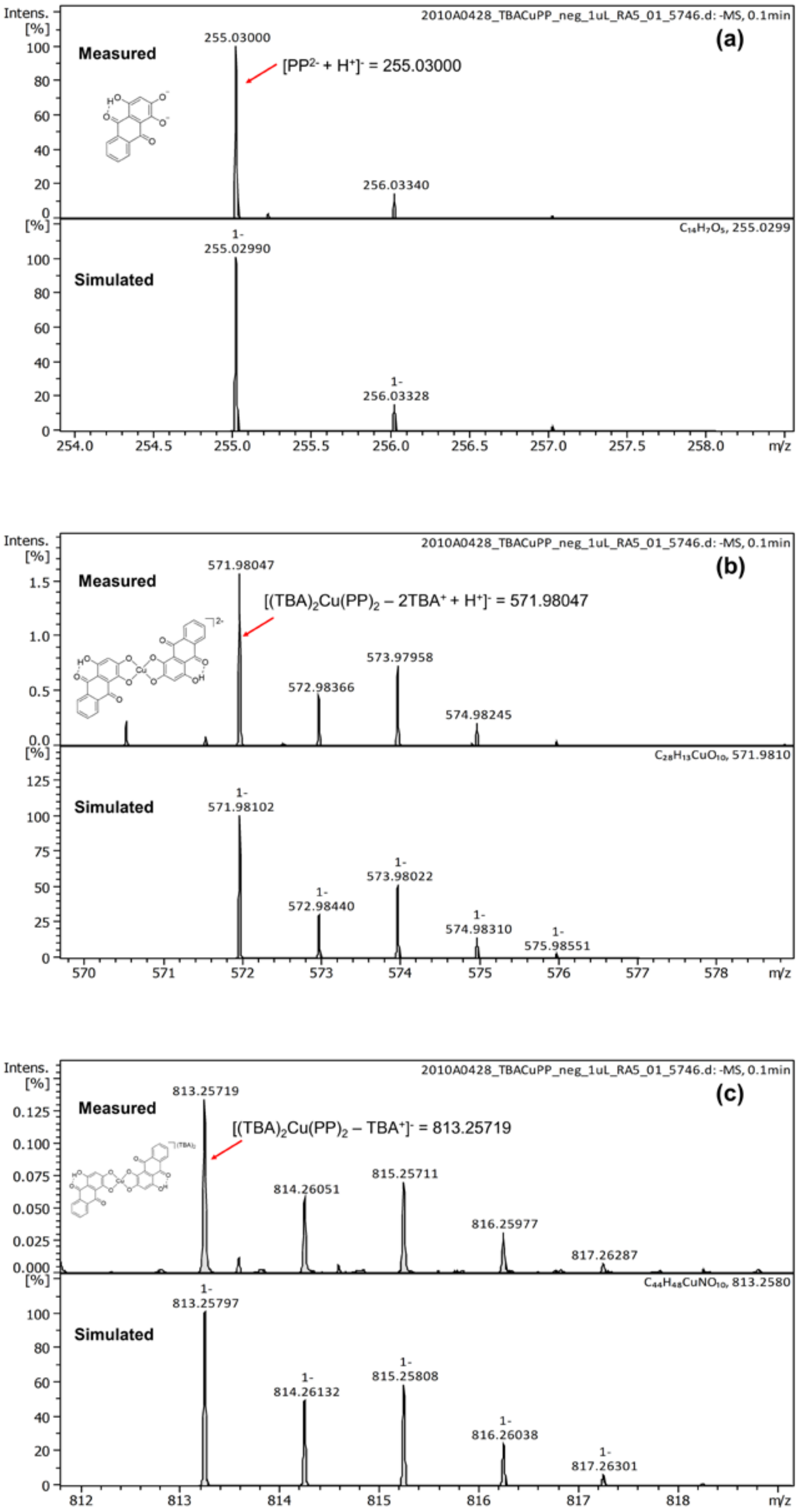

Supplementary Figure 2. HRMS spectra. HRMS spectra (magnification of Supplementary Figure 1b) of $(TBA)_2(CuPP)_2$ in CH_3OH (negative ion mode). The most intense signal is at m/z ($[PP^{2-} + H^+]^-$) = 255.03000 (calcd: 255.02990) (a); A peak at m/z = 571.98047 (calcd: 571.98102) corresponds to a fragment of $[(TBA)_2Cu(PP)_2 - 2TBA^+ + H^+]^-$ (b); A peak at m/z = 813.25719 (calcd: 813.25797) corresponds to of $[(TBA)_2Cu(PP)_2 – TBA^+]^-$ (c).

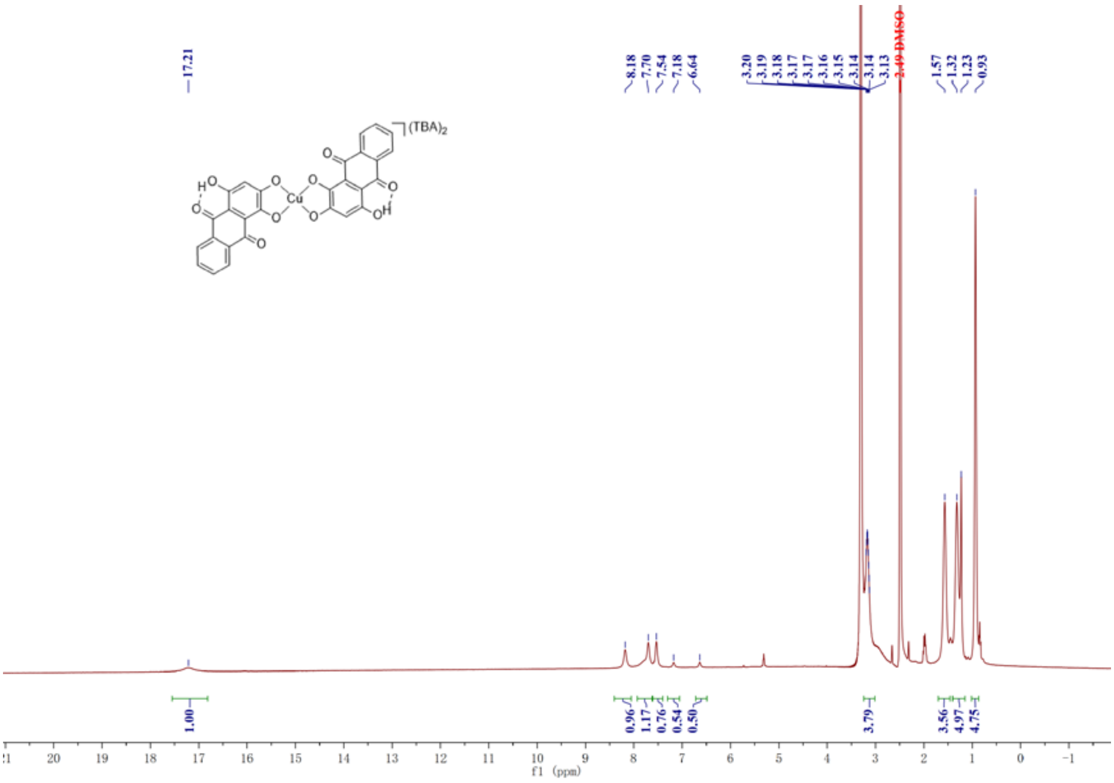

Supplementary Figure 3. ¹H NMR spectrum. ¹H NMR spectrum of CuPP in d_6-DMSO.

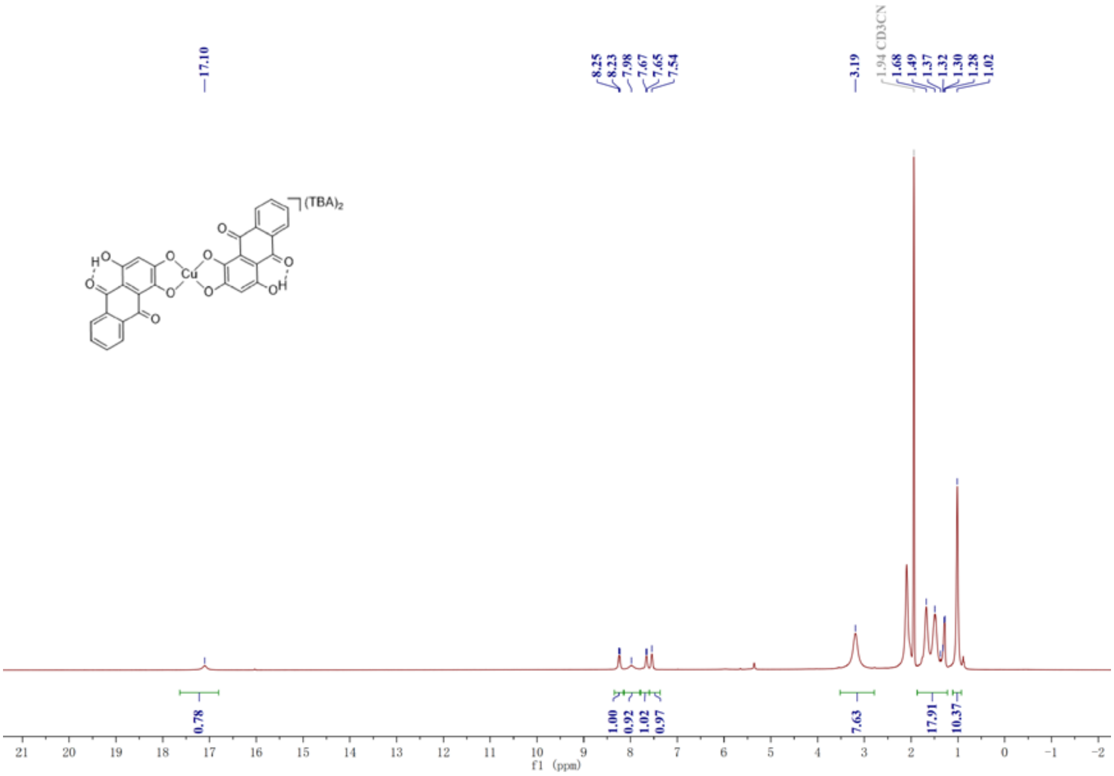

Supplementary Figure 4. ¹H NMR spectrum. ¹H NMR spectrum of CuPP in CD_3CN.

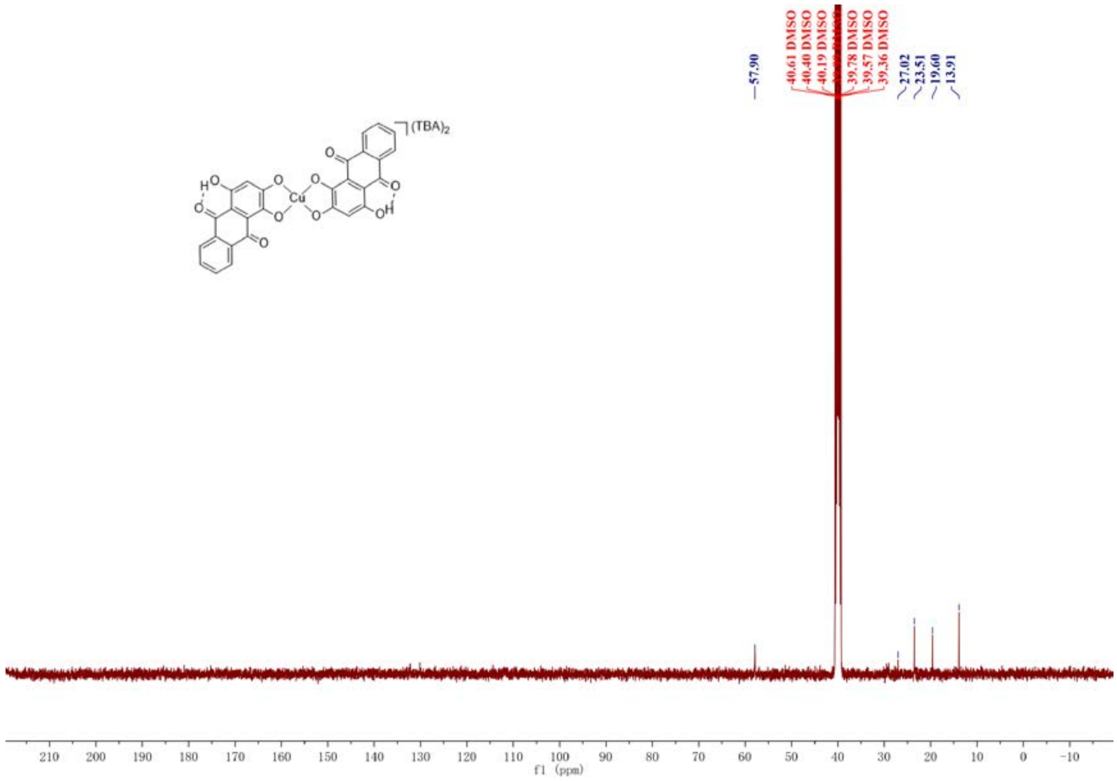

Supplementary Figure 5. ^{13}C NMR spectrum. ^{13}C NMR spectrum of CuPP in d_6-DMSO.

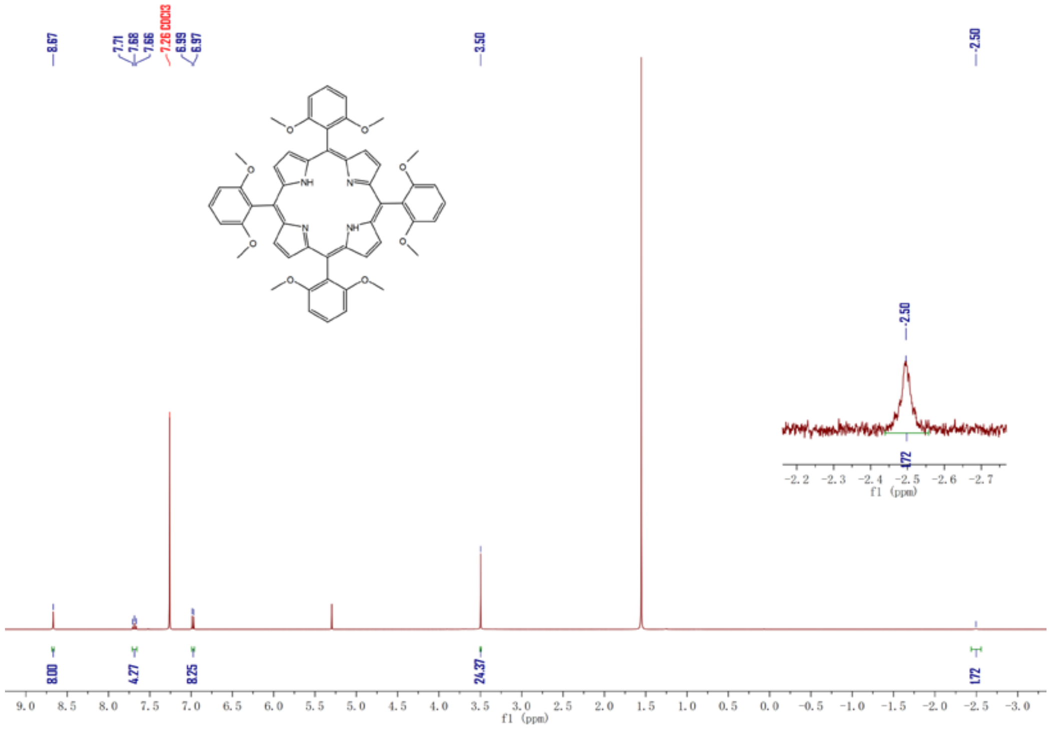

Supplementary Figure 6. ^{1}H NMR spectrum. ^{1}H NMR spectrum of 5, 10, 15, 20-tetrakis(2',6'-dimethoxyphenyl)-21H,23H-porphyrin in $CDCl_3$.

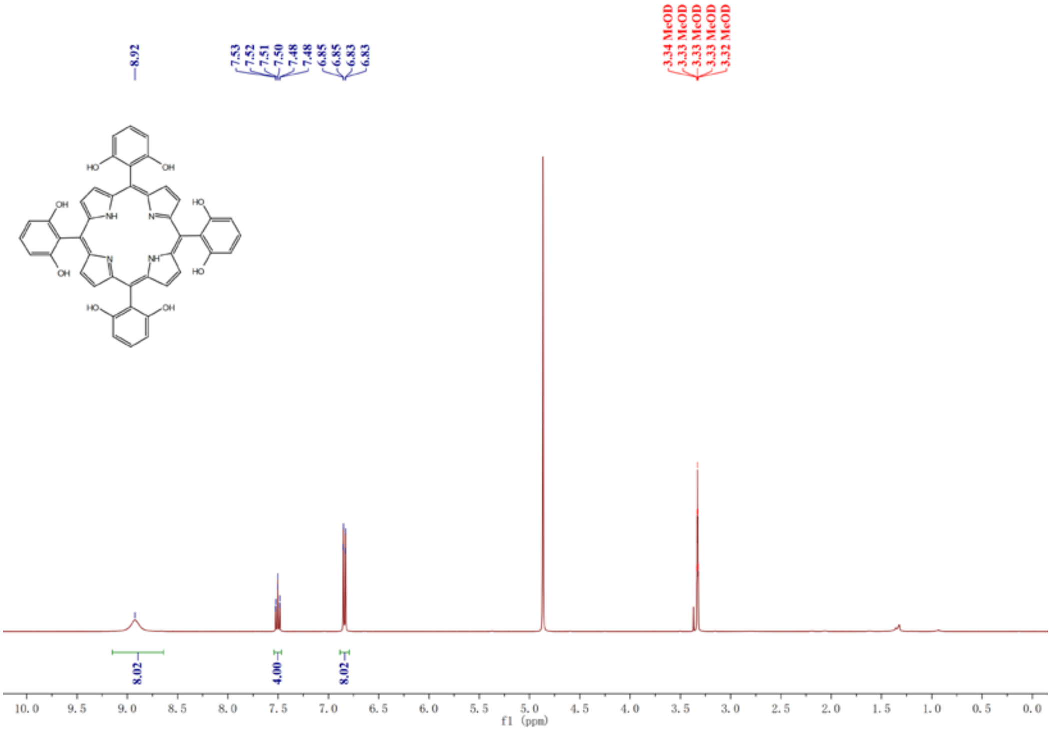

Supplementary Figure 7. ^{1}H NMR spectrum. ^{1}H NMR spectrum of 5, 10, 15, 20-tetrakis(2’,6’-dihydroxyphenyl)-21H,23H-porphyrin in CD_3OD.

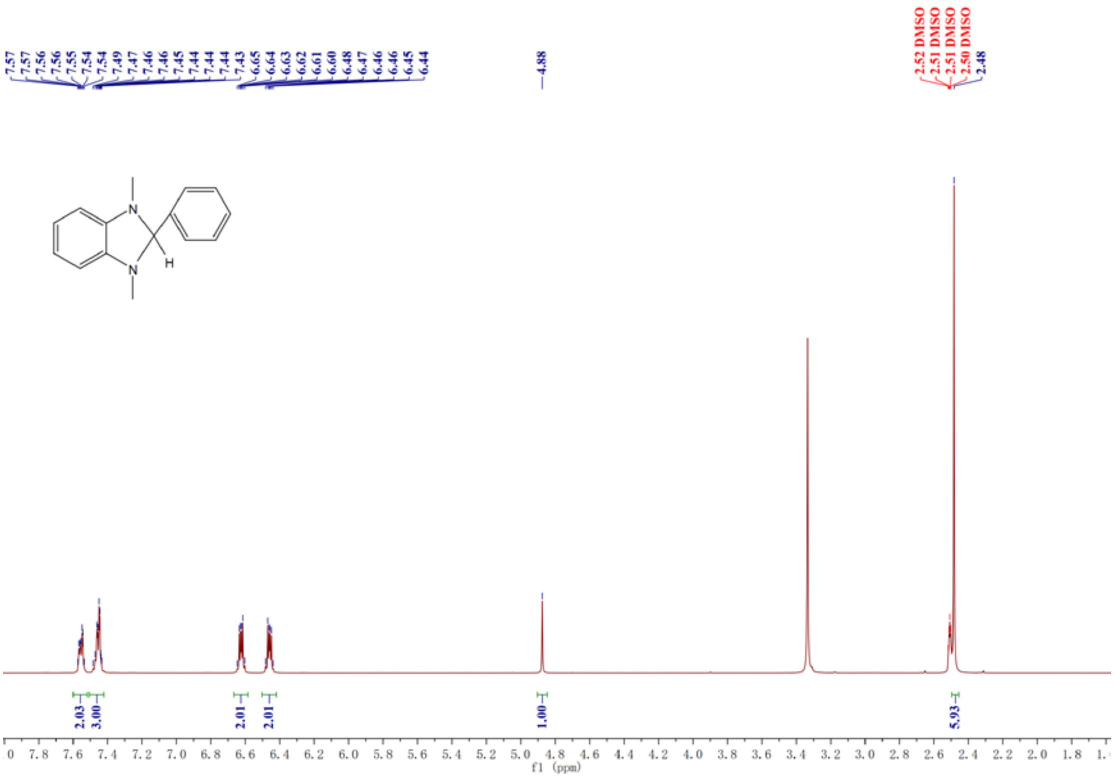

Supplementary Figure 8. ^{1}H NMR spectrum. ^{1}H NMR spectrum of BIH in d_6-DMSO.

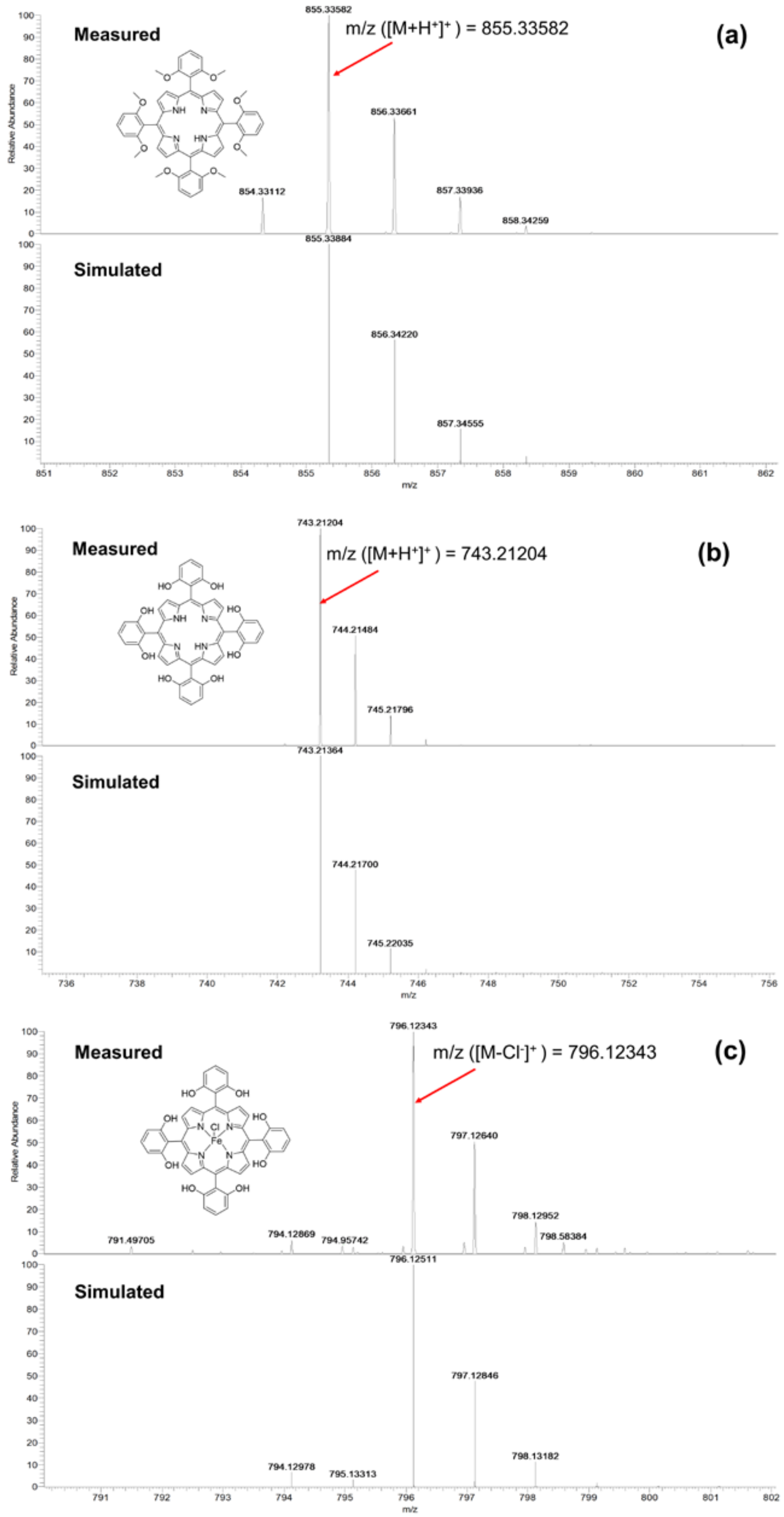

Supplementary Figure 9. HRMS spectra. HRMS spectra of 5, 10, 15, 20-tetrakis(2’,6’-dimethoxyphenyl)-21H,23H-porphyrin (a); 5, 10, 15, 20-tetrakis(2’,6’-dihydroxyphenyl)-21H,23H-porphyrin (b); and chloro iron (III) 5, 10, 15, 20-tetrakis(2’,6’-dihydroxyphenyl)-21H,23H-porphyrin (FeTDHPP) (c) in CH_3OH (positive ion mode).

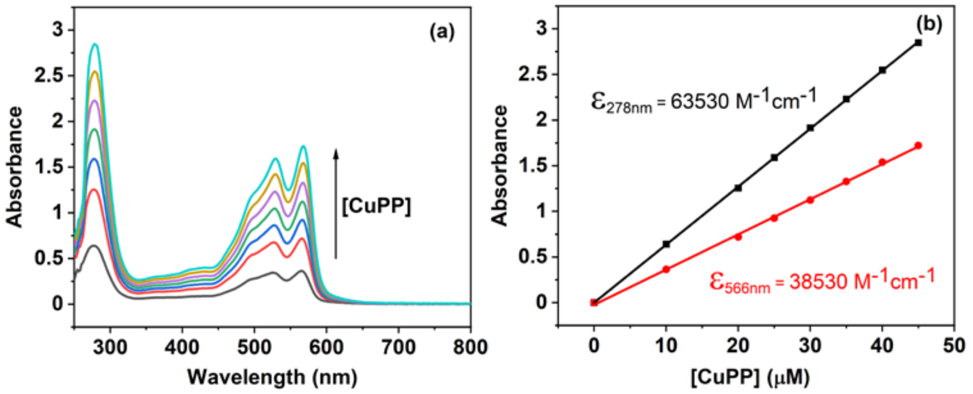

Supplementary Figure 10. UV-vis spectra. UV-vis spectra of CuPP at different concentrations (a); linear plots of absorbance at 278 nm (black) and 566 nm (red) (b).

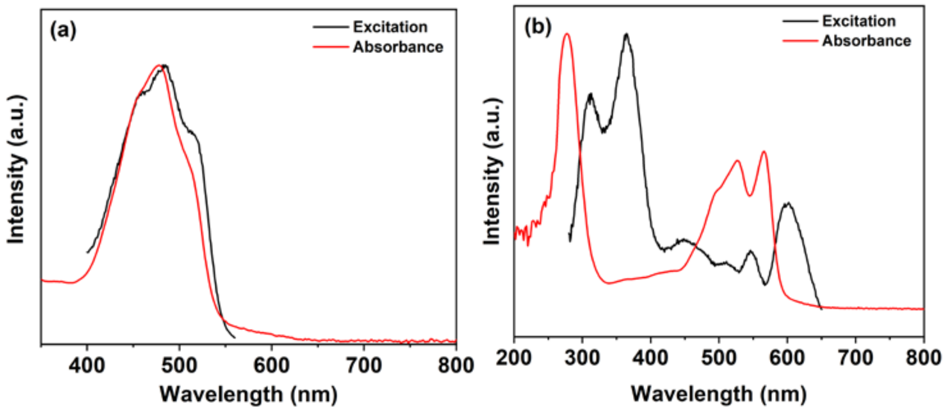

Supplementary Figure 11. Excitation and absorbance spectra. Normalized excitation and absorbance spectra of PP (a) and CuPP (b) at 298 K in DMF.

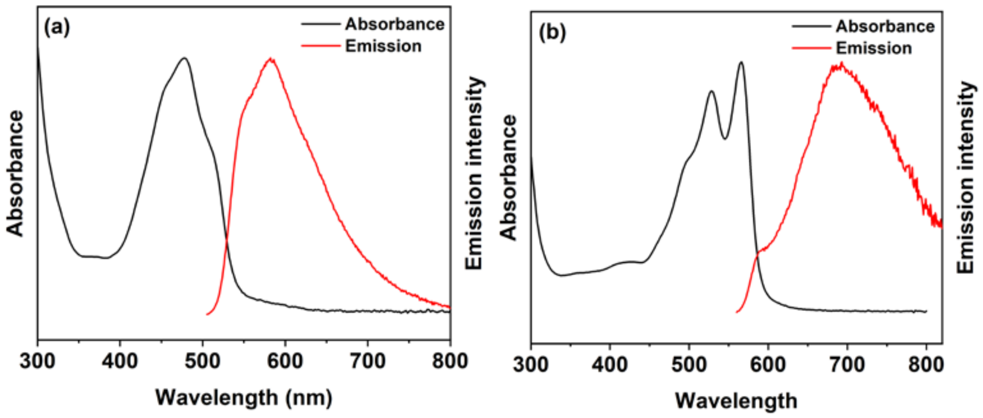

Supplementary Figure 12. Absorbance and emission spectra. Normalized absorbance and emission spectra of PP (a, λ_{exc}: 484 nm) and CuPP (b, λ_{exc}: 540 nm) at 298 K in DMF.

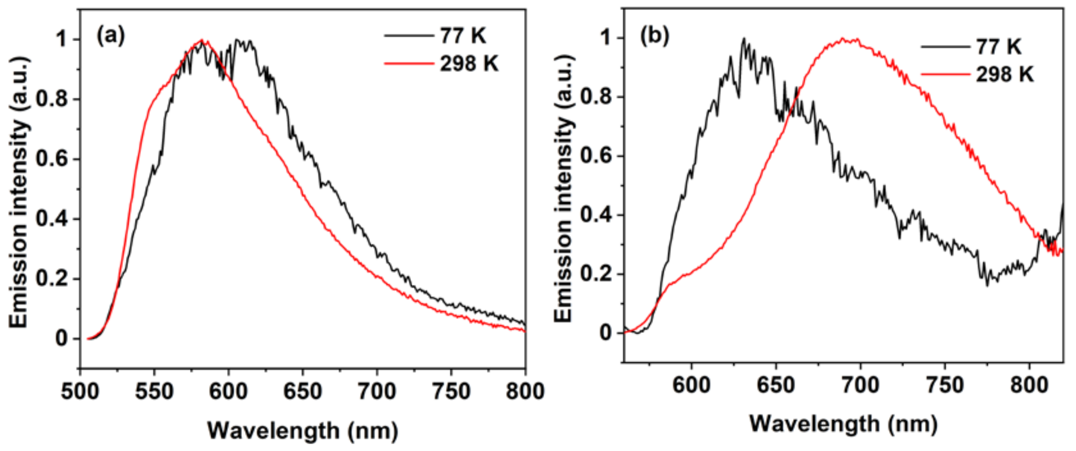

Supplementary Figure 13. Emission spectra. Normalized emission spectra of PP (a, λ_{exc}: 484 nm) and CuPP (b, λ_{exc}: 540 nm) at 298 K and 77K in DMF.

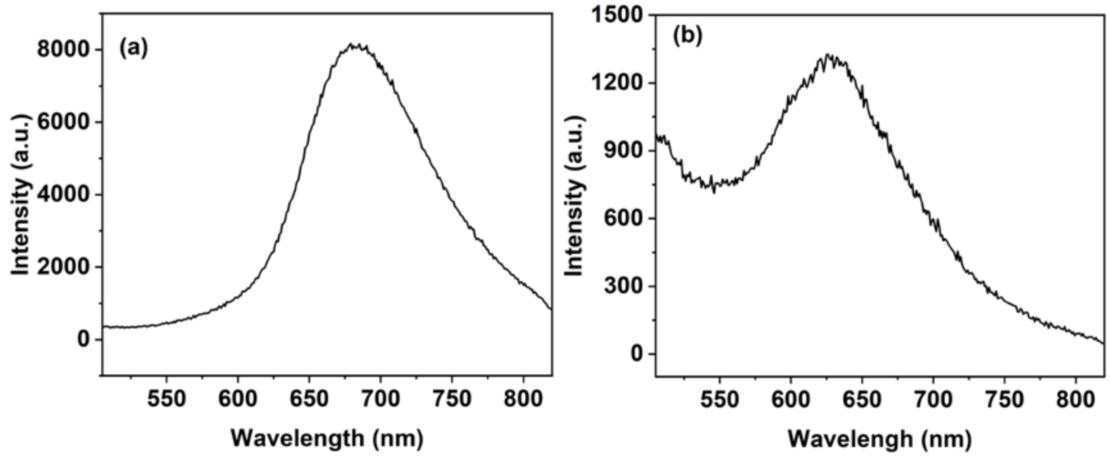

Supplementary Figure 14. Emission spectra. Solid state emission spectra of PP (a) and CuPP (b) with excitation wavelength of 350 nm at 298 K.

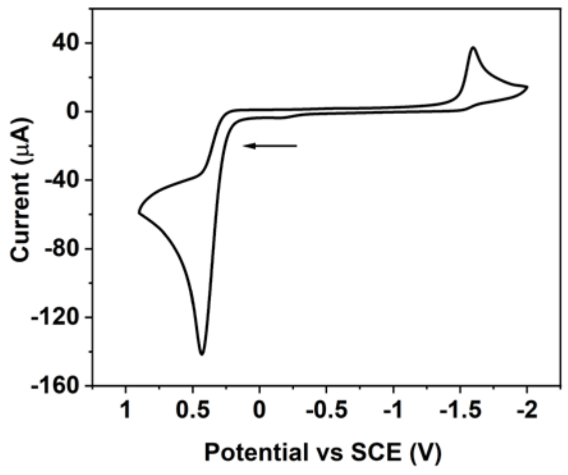

Supplementary Figure 15. Electrochemical study. CV of BIH (5 mM) in DMF containing 0.1M $TBAPF_6$ at 0.1 V/s scan rate.

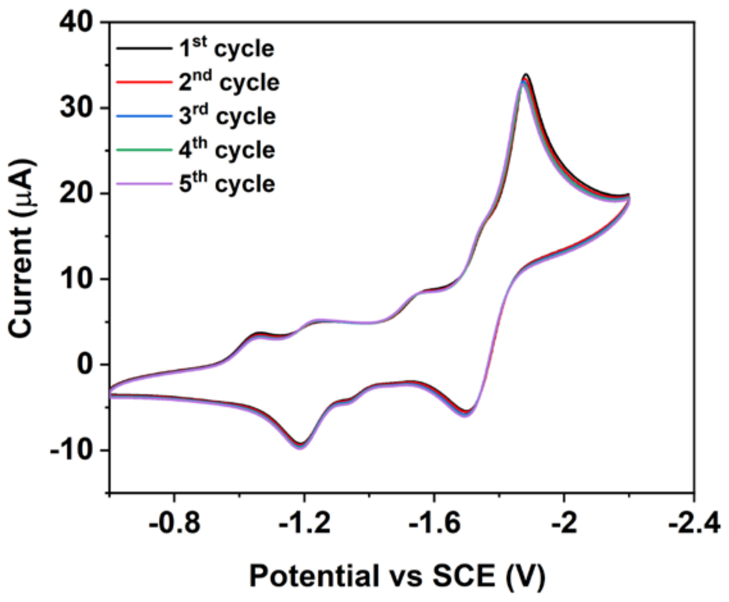

Supplementary Figure 16. Electrochemical study. CV of CuPP (1 mM) with multiple scans in DMF containing 0.1M $TBAPF_6$ at 0.1 V/s scan rate.

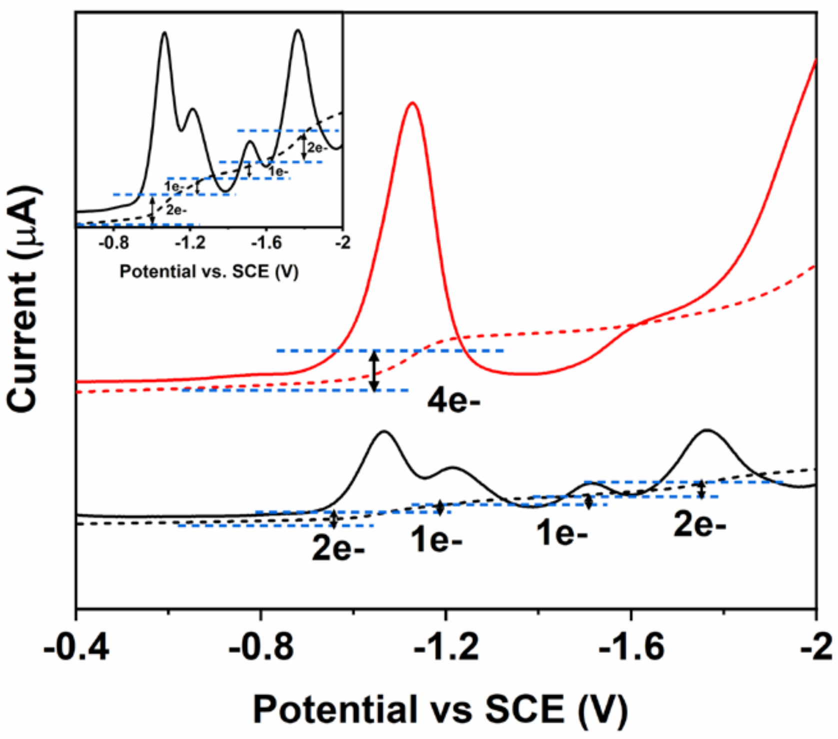

Supplementary Figure 17. Electrochemical study. SWV of 1 mM CuPP under N_2 (black solid) or CO_2 (red solid) in DMF containing 0.1 M $TBAPF_6$ at scan rate 0.1 V/s; dash lines show integrals for reduction waves. Inset: magnification of the SWV under N_2.

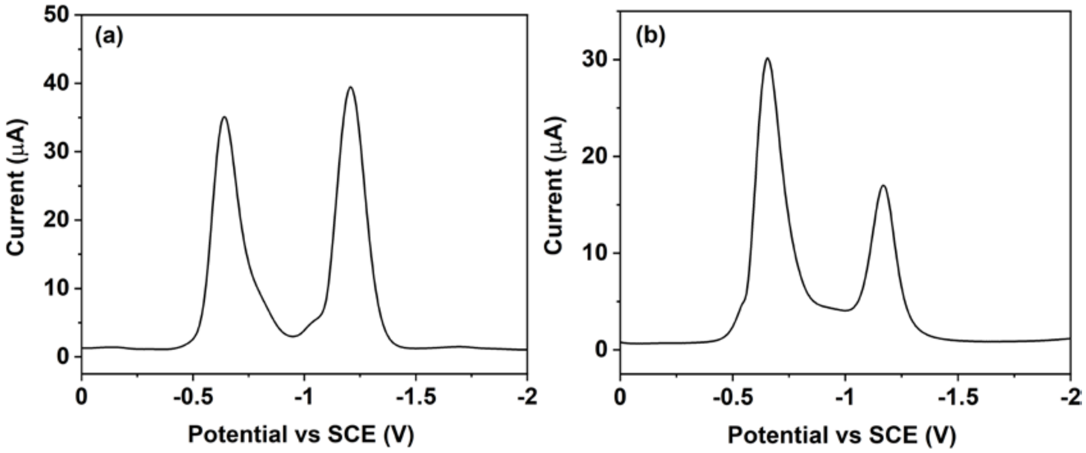

Supplementary Figure 18. Electrochemical study. SWV of 2 mM PP under N_2 (a) or CO_2 (b) in DMF containing 0.1M $TBAPF_6$ at 0.1 V/s scan rate.

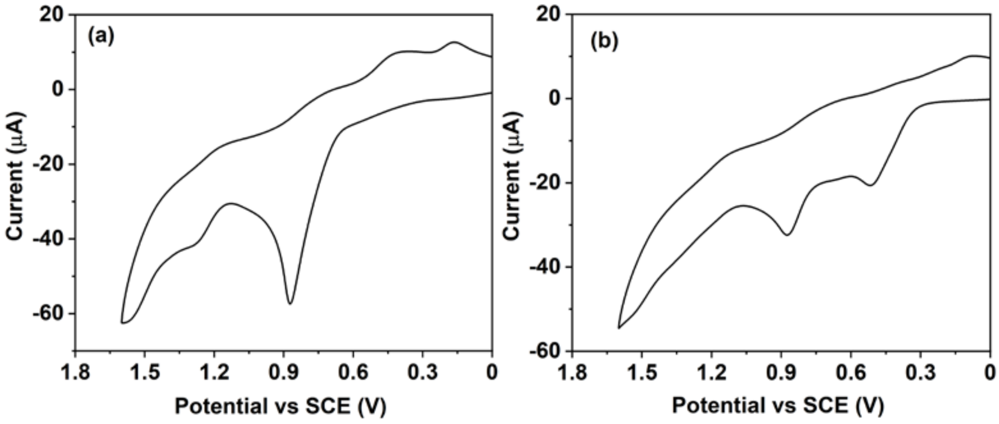

Supplementary Figure 19. Electrochemical study. Oxidative part of CVs of 1 mM PP (a) and 1 mM CuPP (b) in DMF containing 0.1M $TBAPF_6$ at 0.1 V/s scan rate.

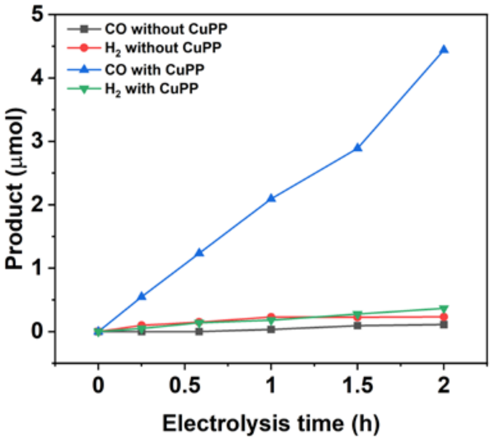

Supplementary Figure 20. Electrocatalytic CO_2 reduction. Bulk electrolysis time course for the amount of CO and H_2. Condition: with or without CuPP (10 μM) under CO_2-saturated DMF containing 0.1M $TBAPF_6$ at -1.89 V (vs SCE) using a carbon rod working electrode.

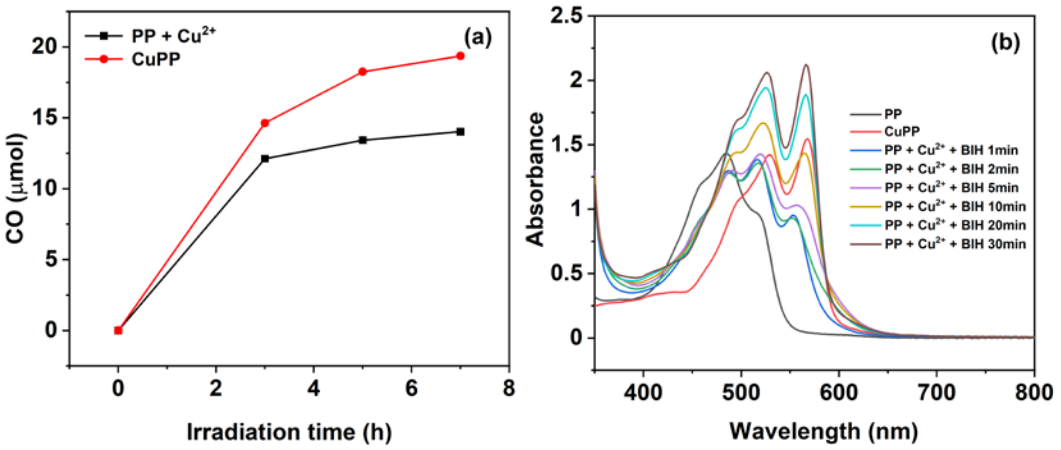

Supplementary Figure 21. Photocatalytic CO_2 reduction and UV-vis spectra changes with the *in situ* generated CuPP. (a) The amount of CO produced with the *in situ* generated CuPP (0.1 mM Cu^{2+} and 0.2 mM PP) (black) or with isolated CuPP (red) as the photosensitizers in CO_2-saturated DMF solution containing 1.0 µM FeTDHPP and 10 mM BIH; (b) UV-vis spectra changes for a solution containing 0.1 mM Cu^{2+}, 0.2 mM PP, and10 mM BIH in DMF. "Source data are provided as a Source Data file."

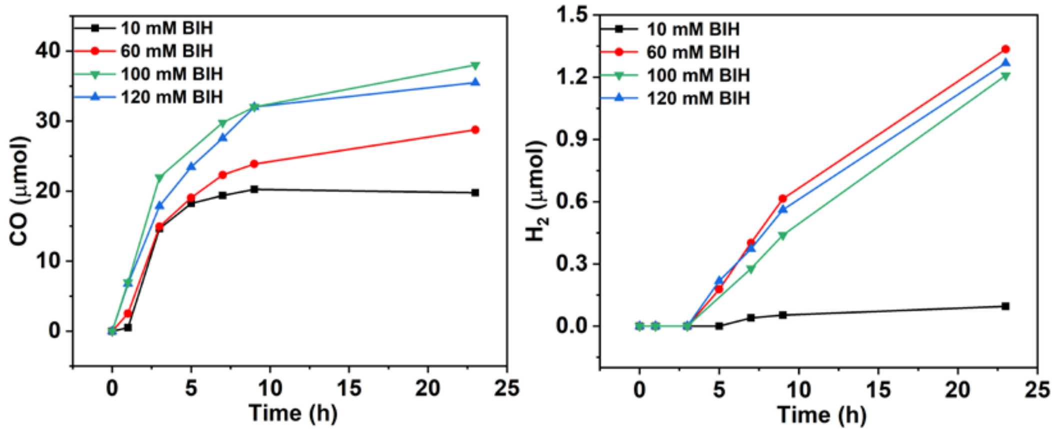

Supplementary Figure 22. Photocatalytic CO_2 reduction. CO (left) and H_2 (right) generation in CO_2-saturated DMF solutions containing 1.0 µM FeTDHPP and 0.1 mM CuPP at different BIH concentrations. "Source data are provided as a Source Data file."

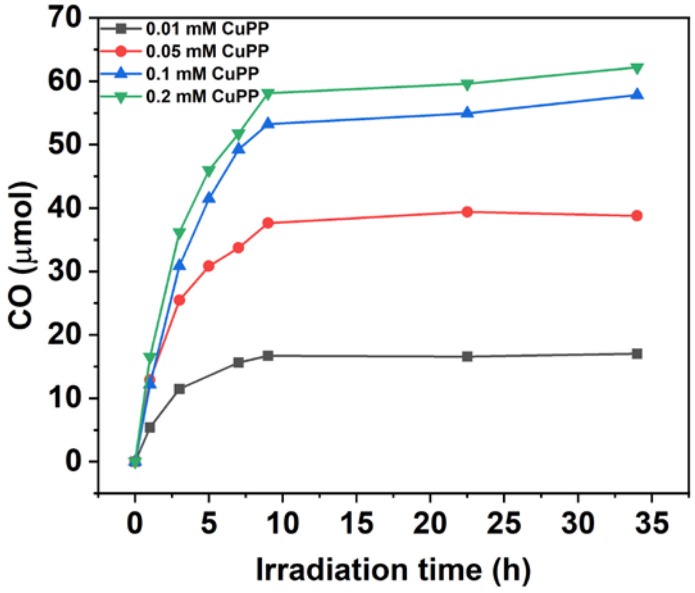

Supplementary Figure 23. Photocatalytic CO_2 reduction. CO generation in CO_2-saturated DMF solutions containing 2 μM FeTDHPP and100 mM BIH at different CuPP concentrations. "Source data are provided as a Source Data file."

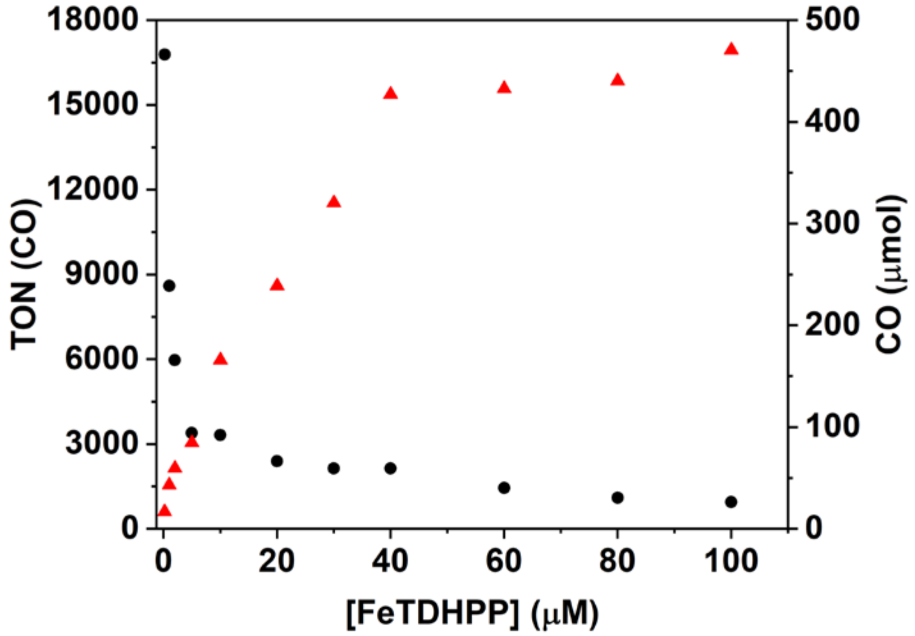

Supplementary Figure 24. Photocatalytic CO_2 reduction. TON (dot) and amounts (triangle) of CO of the photocatalytic CO_2 reduction experiments irradiated for 23h in CO_2-saturated DMF solution containing 0.1 mM CuPP and 100 mM BIH with varying amounts of FeTDHPP. "Source data are provided as a Source Data file."

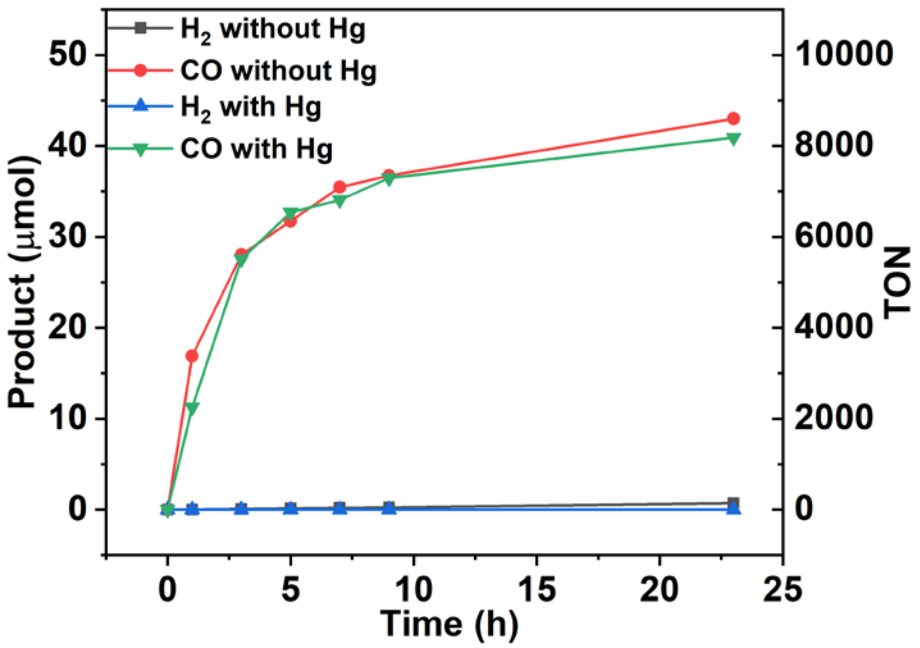

Supplementary Figure 25. Photocatalytic CO_2 reduction. Photocatalytic CO_2 reduction in the presence (0.02 mL, ~270000 eq. vs. catalyst) and absence of Hg in CO_2-saturated DMF solution containing 0.1 mM CuPP, 1.0 μM FeTDHPP, and 100 mM BIH. "Source data are provided as a Source Data file."

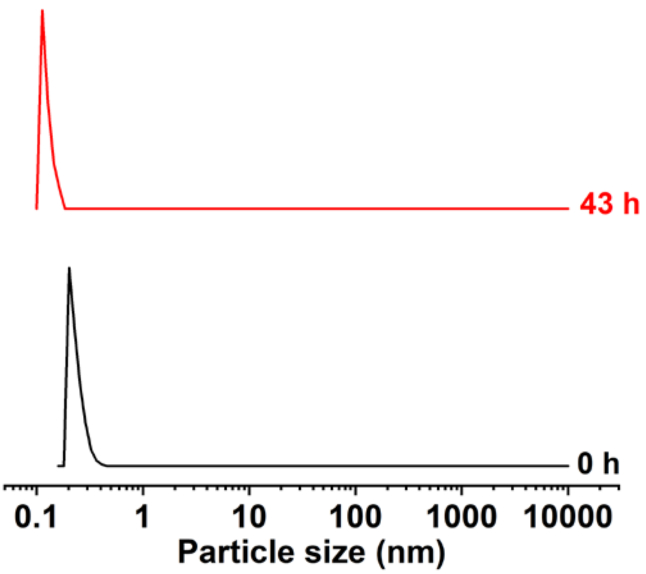

Supplementary Figure 26. Dynamic light scattering (DLS) measurement. Particle size distribution of a CO_2-saturated DMF solution containing 0.1 mM CuPP, 1.0 μM FeTDHPP, and 100 mM BIH determined by dynamic light scattering (DLS) before and after irradiation.

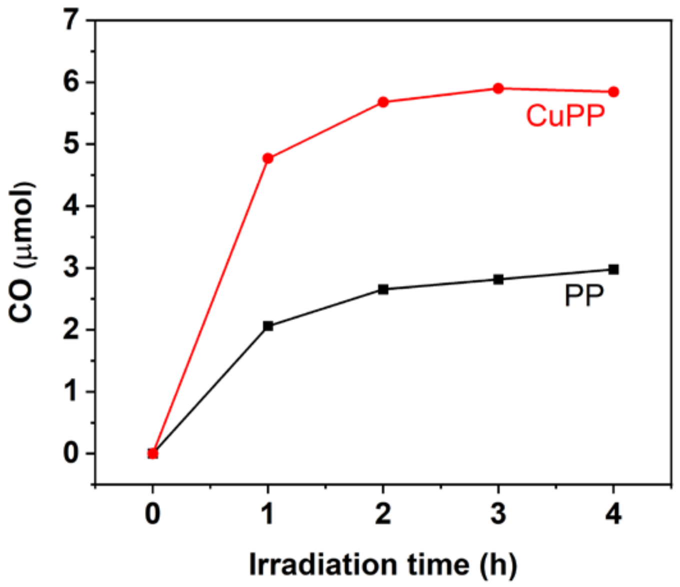

Supplementary Figure 27. Photocatalytic CO_2 reduction. Photocatalytic CO_2 reduction in CO_2-saturated DMF solutions containing: 0.2 mM PP (black) or 0.1 mM CuPP (red), 2 μM Co(qpy)Cl_2 (qpy = 2,2':6',2":6",2'"-quaterpyridine)[1] and 30 mM BIH. "Source data are provided as a Source Data file."

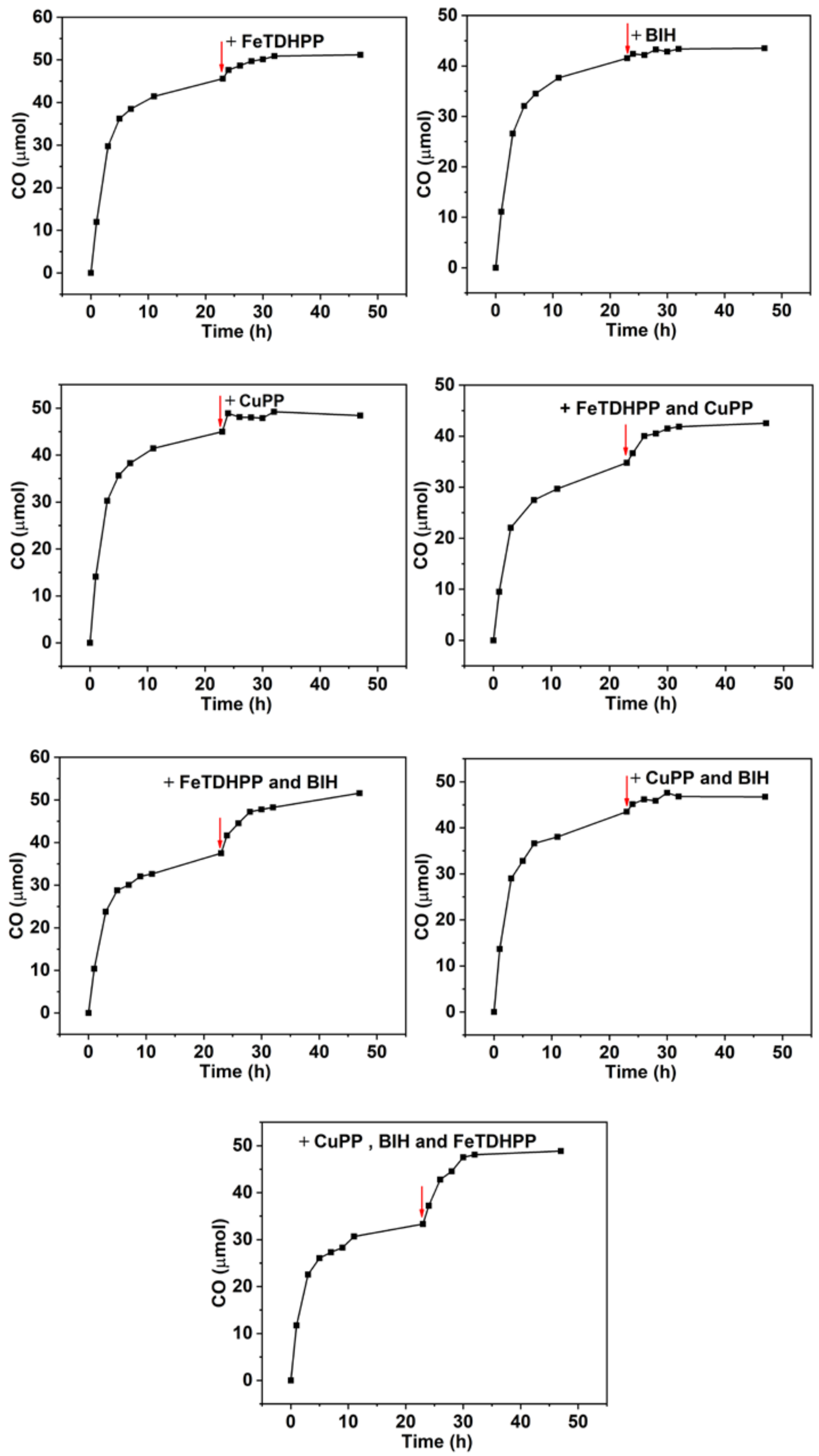

Supplementary Figure 28. Stability tests of photocatalytic systems. Stability tests of photocatalytic systems containing 0.1 mM CuPP, 1 μM FeTDHPP, and 100 mM BIH. The same amount of each component or their mixtures were added to reaction vails at 23 hours. "Source data are provided as a Source Data file."

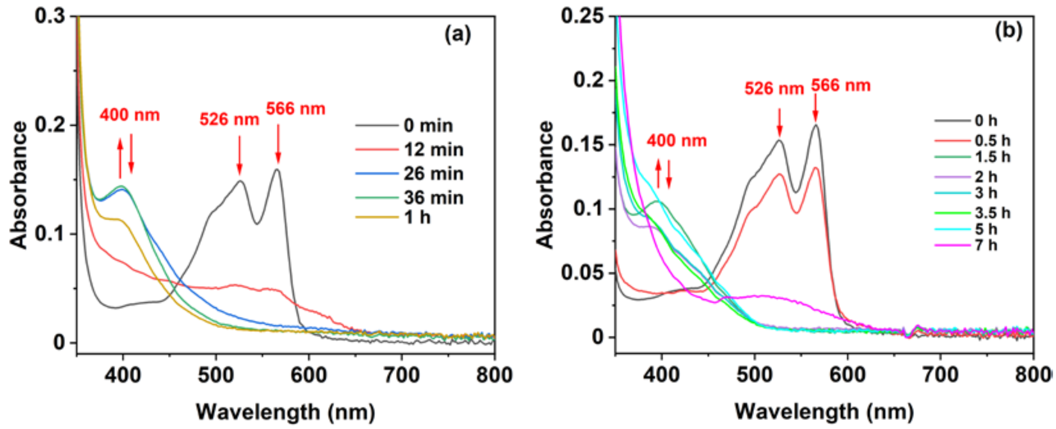

Supplementary Figure 29. UV-vis absorption spectra. UV-vis absorption spectra of systems containing: (a) 0.1 mM CuPP, and 100 mM BIH; (b) 0.1 mM CuPP, 0.2 μM FeTDHPP and 10 mM BIH upon irradiation with white LED light in a 2 mm path length of quartz cuvette (dilution factor of 5).

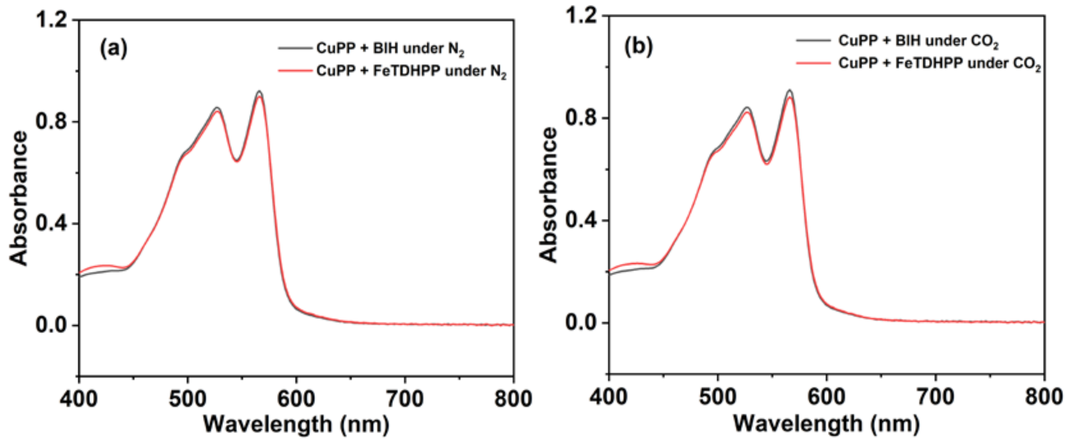

Supplementary Figure 30. UV-vis absorption spectra. UV-vis absorption spectra of CuPP (25 μM) with addition of BIH (10 mM) or FeTDHPP (1 μM) under N_2 (a) or CO_2 (b).

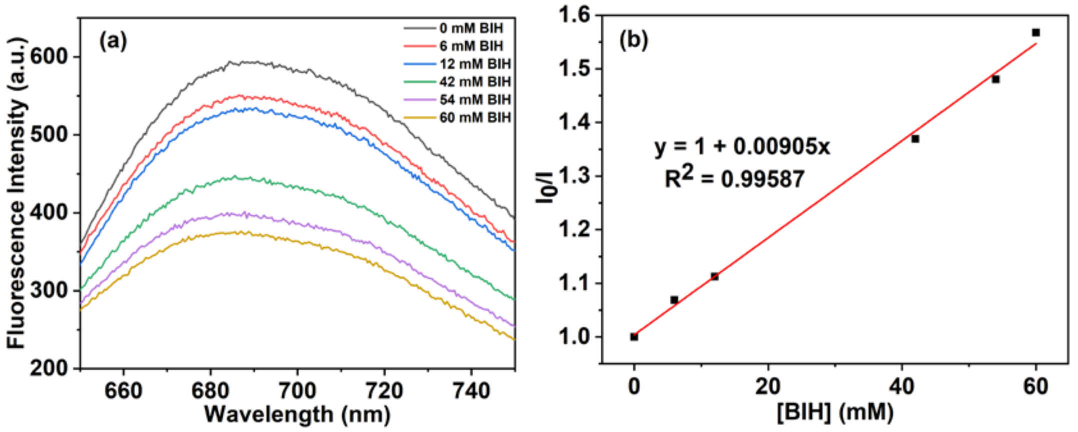

Supplementary Figure 31. Emission quenching. Stern-Volmer plot (b) of the emission quenching (a) (λ_{exc}:540 nm) of CuPP (25 µM) by BIH in DMF. "Source data are provided as a Source Data file."

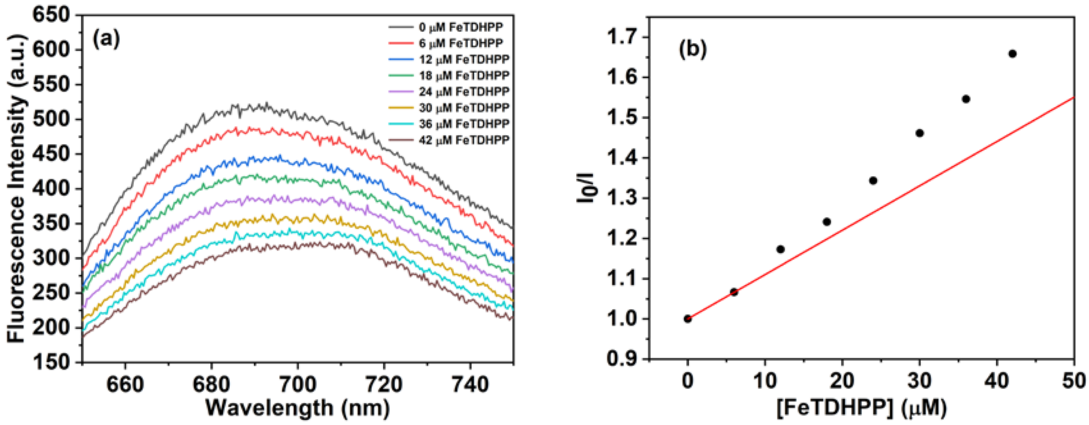

Supplementary Figure 32. Emission quenching. Stern-Volmer plot (b) of the emission quenching (a) (λ_{exc}:540 nm) of CuPP (25 µM) by FeTDHPP**.** "Source data are provided as a Source Data file."

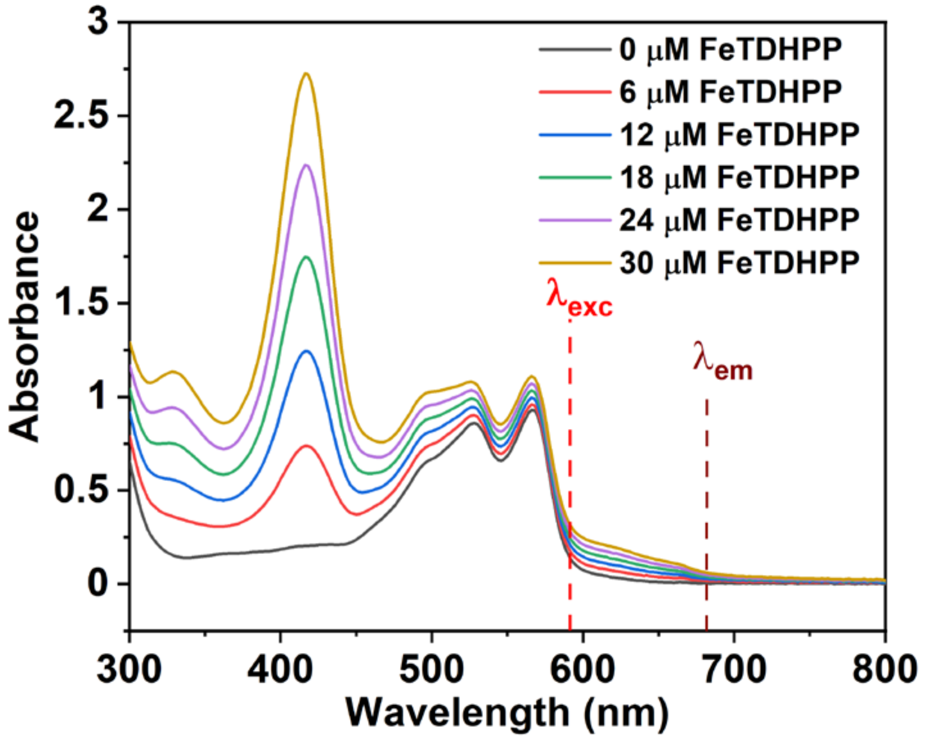

Supplementary Figure 33. UV-vis absorption spectra. Absorption spectra of CuPP (25 μM) with addition of FeTDHPP.

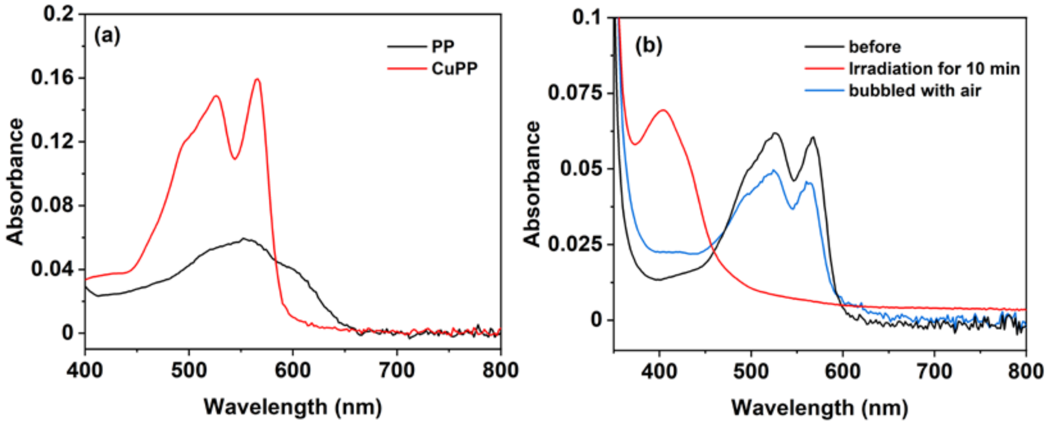

Supplementary Figure 34. UV-vis absorption spectra. UV-vis absorption spectra of systems containing: (a) 0.1 mM CuPP (red) or 0.2 mM PP (black), 0.04 μM FeTDHPP and 20 mM BIH (spectra taken with dilution factor of 5); (b) 0.1 mM CuPP, 0.2 μM FeTDHPP and 100 mM BIH before irradiation (black), irradiation for 10 minutes (red) then bubbled with air (blue) (spectra taken with dilution factor of 50). Condition: (a) 2 mm path length quartz cuvette; (b) 1 cm path length quartz cuvette.

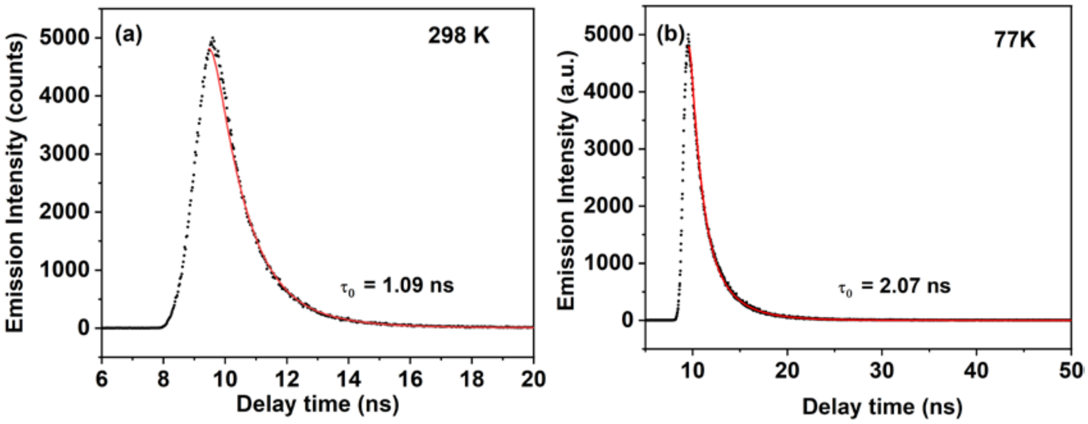

Supplementary Figure 35. Emission decay. Emission decay of PP (50 µM) in DMF at 298K (a) or 77K (b).

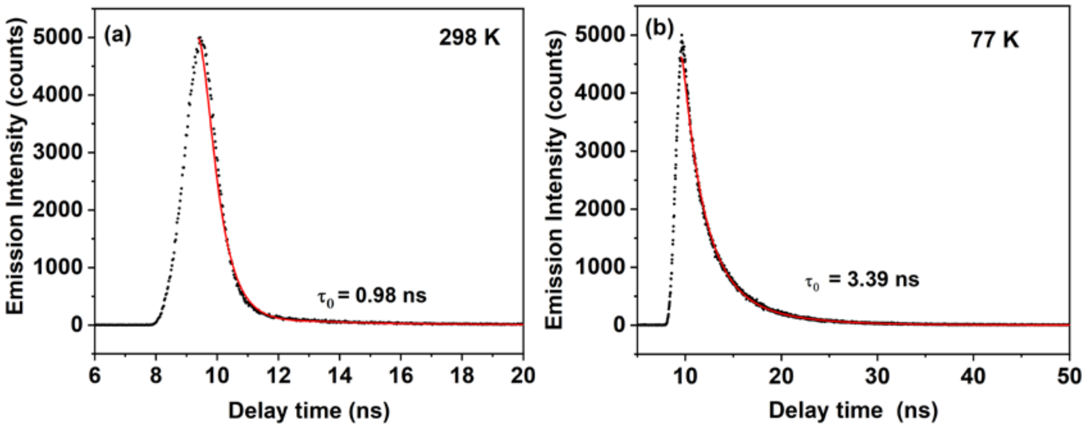

Supplementary Figure 36. Emission decay. Emission decay of CuPP (25 µM) in DMF at 298K (a) and 77K (b).

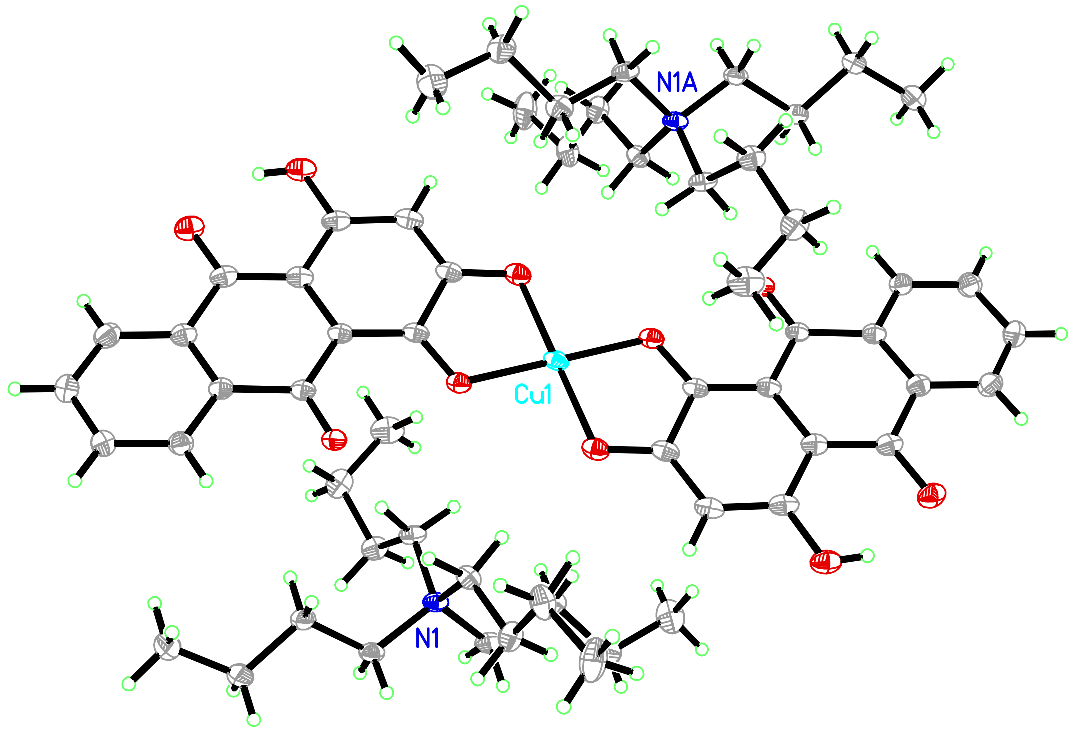

Supplementary Figure 37. Crystal structure of CuPP. Complete ORTEP diagram of CuPP at 50% probability.

Supplementary Table 1. Cu-O bond lengths (Å) of Cu catecholate complexes

Cu-Complex	Cu-O1	Cu-O2	Reference
CuPP	1.9268(17)[a]	1.9168(18)[a]	This work
bis(catecholato)Cu	1.9165(4)[a]	1.9303(3)[a]	[2]
$Cu(py)_2(Lw)_2$	2.454(2)[b]	1.945(2)[c]	[3]
$Cu(Lw)_2(H_2O)_2$	2.336(2)[b]	1.954(3)[a]	[4]
$Cu(L1)_2(EtOH)_2$	2.225(2)[b]	1.9301(17)[a]	[5]
$Cu(Lap)_2(DMF)_2$	2.301(1)[b]	1.914(1)[a]	[6]
CuL_2py_2	2.415(4)[b]	1.948(2)[c]	[7]

[a]phenoxy coordination; [b]quinonic carbonyl coordination; [c]enolic coordination;

Supplementary Table 2. Summary of photophysical data of PP and CuPP. Photophysical parameters of PP and CuPP at 298 K in DMF solutions

Sample	Medium	Absorbance		Fluorescence				
		λ_{max}(nm)	$\varepsilon_{\lambda max}$ (M^{-1} cm^{-1})	λ_{max}(nm)	Φ_F[a]	τ (ns)	$\kappa_F/10^7$ (s^{-1})	$\kappa_{NF}/10^9$ (s^{-1})
PP	DMF	478	9940	582[b]	2.7×10^{-2}	1.1	2.5	0.88
CuPP	DMF	566	38530	693[c]	8.2×10^{-3}	0.98	0.84	1.01

[a] Measured under N_2, by absolute method using an integrating sphere, error 1-20 %. [b] λ_{exc} = 484 nm; [c] λ_{exc} = 540 nm.

Supplementary Table 3. Thermodynamic driving force for electron transfer of photocatalytic systems

Photosensitizers	E_{red} / V	$E_{0,0}$ / eV	E^{*}_{red} / V	E^{*}_{ox} / V	ΔG / eV
PP	-1.21	2.34	1.13	-1.47	-0.8
CuPP	-1.75	2.12	0.37	-1.61	-0.04

$E_{0\text{-}0}$ values were determined from the intersection of the normalized absorption and emission spectra of the CuPP, in CO_2-saturated DMF solution, and converted to eV[8]. The ground state redox potentials (E_{ox} and E_{red}) were measured by electrochemical methods (CVs). The excited state redox potentials were obtained as follows: ESOP (Excited State Oxidation Potential) = $E_{ox}(CuPP^{*}) = E_{ox} - E_{0\text{-}0}$; ESRP (Excited State Reduction Potential) = $E_{red}(CuPP^{*}) = E_{red} + E_{0\text{-}0}$. The thermodynamic driving force for electron transfer were calculated from Rehm-Weller equation: the difference between reduction potential of excited state of photosensitizer and oxidation potential of BIH as sacrificial reagent. ($\Delta G = E^{0}_{(D+/D)} - E^{0}_{(A/A-)} - E_{0,0} - e^{2}/\varepsilon d$). The last term which represents the columbic attraction energy was neglected because of small contribution to the overall energy. Therefore, the equation was simplified to $\Delta G = E_{ox}(BIH) - E^{*}_{red}(CuPP)$ where $E_{ox}(BIH)$ was +0.33 V (vs SCE). Potentials are given versus SCE.

Supplementary Table 4. Control experiments for photocatalytic CO_2 reduction. Control experiments for photocatalytic CO_2 reduction in a 5 mL CO_2-saturated DMF solution containing FeTDHPP (1 μM), CuPP (0.1 mM)， and BIH (100 mM), under irradiation with white LED light for 23h at 25 ^oC.

Entry[a]	FeTDHPP (μM)	CO (μmol)	H_2 (μmol)	TON_{CO}	TON_{H2}	Selectivity to CO
1	1	43	0.72	8600	144	98%
2	0	2.2	0	-	-	0
3	1	0	0	0	0	0
4	1	0	7.3	0	1466	0
5	1	0	0	0	0	0
6	1	0	0	0	0	0
7	0.2	16.1	0.84	16109	843	95%
8	2	59.6	0.63	5963	63	99%
9	1	41	0	8183	0	100%
10	5	84.6	1.08	3385	43	99%
11	10	156.5	0.62	3131	12	99%

[a] Entry 1: under CO_2 atmosphere, Entry 2: without FeTDHPP, Entry 3: without CuPP, Entry 4: under N_2 atmosphere, Entry 5: Experiments were carried out in the dark, Entry 6: without BIH; Entry 7: 0.2 μM FeTDHPP, Entry 8: 2 μM FeTDHPP, Entry 9: added 0.02 mL Hg, Entry 10: 5 μM FeTDHPP, Entry 11: 10 μM FeTDHPP.

Supplementary Table 5. The performance of photocatalytic CO_2 reduction with molecular complexes containing copper complexes photosensitizers in noble-metal-free systems in the literature.

**Photosensitizer**	**Catalyst**	**Solvent**	**Electron donor**	**Initial TOF_{CO} (h^{-1})**	**TON_{CO}**	**TON_{H2}**	**Sel_{CO} (%)[a]**	**Light source**	**Reference**
CuPP (PP = purpurin)	FeTDHPP (0.2 µM)	DMF	BIH	7650	16109	843	95	white LED (λ > 400 nm)	**This work**
in-situ formed [Cu(xantphos)(bathocuproine)]$^+$	Mn(pyrox)$(CO)_3$Br (0.01 µmol)	CH_3CN/TEOA (5:1, v/v)	BIH	-	1058	0	100	Hg lamp (λ > 415 nm)	9
Cu (P^P-$(SO_3Na)_2$)(N^N$(SO_3Na)_2$)]BF_4	CoTMPyP (10 µM)	H_2O	AscHNa	2600	2680	820	77	Xe lamp (λ > 400 nm)	10
in-situ formed [Cu(xantphos)(bathocuproine)]$^+$	{[Fe^{III}(LN4H_2)Cl]$_2$(µ -O)}$^{2+}$ (25 µM)	DMF/TEOA (4:1, v/v)	BIH	114	565	116	84	Xe lamp (λ > 400 nm)	11
[Cu_2(P_2bph)$_2$]$^{2+}$	fac-Mn(X_2bpy)$(CO)_3$Br (50 µM)	DMA/TEOA (4:1, v/v)	BIH	-	1004	68	95	Hg lamp (λ > 436 nm)	12
in-situ formed [Cu(xantphos)(bathocuproine)]$^+$	Fe^{II} cyclopentadienone (130 µM)	NMP/TEOA (5:1, v/v)	BIH	-	487	7	99	Hg lamp (λ = 400 – 700 nm)	13
Cu(dmp)$(P)_2^+$	Fe$(dmp)_2(NCS)_2$ (50 µM)	CH_3CN/TEOA (5:1, v/v)	BIH	-	273	75	78	Hg lamp (λ = 436 nm)	14

[a]Sel_{CO}: seletivity of CO production among all possible CO_2 reduction products

Supplementary Table 6. The performance of photocatalytic CO_2 reduction with molecular complexes containing organic photosensitizers in noble-metal-free systems in the literature

Photosensitizer	Catalyst	Solvent	Electron donor	Initial TOF_{CO} (h^{-1})	TON_{CO}	TON_{H2}	Sel_{CO}(%)[a]	Light source	Reference
9-CNA	FeTDHPP (2 μM)	MeCN	TEA	-	60	0	100	Xe lamp (λ > 400 nm)	15
Non-sensitized	Fe-*p*-TMA (2 μM)	MeCN	BIH	-	101	0	100	solar simulator, 1 sun (λ > 420 nm)	16
Non-sensitized	FeTDHPP (10 μM)	MeCN	TEA	6.3	30	10	75	Xe lamp (cut off IR and low UV)	17
3,7-di(4-biphenyl)-1-naphthalene-10-phenoxazine	Fe-*p*-TMA (10 μM)	DMF	TEA	-	140	23	73	solar simulator, 1 sun (λ > 435 nm)	18
Purpurin	Fe-*p*-TMA (2 μM)	MeCN/H_2O (1:9, v/v)	TEA	-	60	3	95	solar simulator, 1 sun (λ > 420 nm)	19
Purpurin	$[Fe(dqtpy)(H_2O)]^{2+}$ (50 μM)	DMF	BIH	-	544	4	99.3	blue LED (λ = 460 nm)	20
Purpurin	$[Fe(qpy)(H_2O)_2]^{2+}$ (5 μM)	DMF	BIH	-	1365	0	92	blue LED (λ = 460 nm)	1
Purpurin	$[Co(qpy)(H_2O_2]^{2+}$ (5 μM)	DMF	BIH	-	790	11	90		
ZnTPP	*fac*-[Mn(phen)(CO)Br] (50 μM)	MeCN/H_2O (20:1,v/v)	TEA	-	119	0	86	Xe lamp (500 W)	21
4CzIPN	FeTotpy (10μM)	DMF/H_2O (3:2, v/v)	TEA	3600	2250	16	99.3	white LEDs (λ = 420-650 nm)	22
p-terphenyl	Co^{II}[TBPc] (20 μM)	MeCN	TEA	-	50	-	100	Xe lamp (λ > 310 nm)	23

[a]Sel_{CO}: seletivity of CO production among all possible CO_2 reduction products

Supplementary Table 7. The performance of photocatalytic CO_2 reduction with molecular complexes containing nanostructured photosensitizers in noble-metal-free systems in the literature

**Photosensitizer**	**Catalyst**	**Solvent**	**Electron donor**	**Initial TOF_{CO} (h^{-1})**	**TON_{CO}**	**TON_{H2}**	**Sel_{CO}(%)[a]**	**Light source**	**Reference**
mpg-C_3N_4	$[Fe(qpy)(H_2O)_2]^{2+}$ (20 μM)	MeCN/TEOA (4:1, v/v)	TEOA	-	155	<1	97	Hg lamp (λ ≥ 400 nm)	24
$CuInS_2$/ZnS	Fe-*p*-TMA (1 μM)	H_2O	TEOA	-	450	4.5	99	monochromati-c laser pointer (λ = 450 nm)	25
CdS-MPA	Dinuclear cobalt complex (1 μM)	H_2O	TEOA	-	1380	32	95	Xe lamp (λ > 420 nm)	26
ZnSe	$Ni(cyclam)Cl_2$ (10 μM)	H_2O	$AcsH_2$	-	283	549	34	solar simulator, 1 sun (λ > 400 nm)	27
CdS	$[Ni(terpyS)_2]^{2+}$ (100 μM)	H_2O	TEOA	-	20	n.r.	93	solar simulator, 1 sun (λ > 400 nm)	28
$CuInS_2$/ZnS	FeTPP (2 μM)	DMSO	TMPD	-	60	10	84	monochromati-c laser pointer (λ = 450 nm)	29

[a]Sel_{CO}: seletivity of CO production among all possible CO_2 reduction products

Supplementary Table 8. Homogeneous photocatalytic CO_2 reduction system containing porphyrin complex and noble metal photosensitizer

Photosensitizer	Catalyst	Solvent	Electron donor	Initial TOF_{CO} (h^{-1})	TON_{CO}	TON_{H2}	Sel_{CO} (%)[a]	Light source	Reference
$Ru(bpy)_3Cl_2$	CoTPPS (0.5 µM)	H_2O	AscHNa	2400	4000	5756	41	Xe lamp (λ > 400 nm)	30
fac-$Ir(ppy)_3$	FeTDHPP (2 µM)	MeCN	TEA	-	140	11	93	Xe lamp (λ > 420 nm)	15
fac-$Ir(ppy)_3$	FeTDHPP (2 µM)	MeCN	TEA	-	139	15	77	Solar simulator(λ > 420 nm)	31
fac-$Ir(ppy)_3$	Fe-*p*-TMA (2 µM)	MeCN	TEA	-	367	26	78	Solar simulator(λ > 420 nm)	31
$Ir(ppy)_2(bpy)$	Fe-*p*-TMA (2 µM)	MeCN	TEA	-	178	103	57	Solar simulator(λ > 420 nm)	32

[a]Sel_{CO}: seletivity of CO production among all possible CO_2 reduction products

Supplementary Table 9. Crystal data and structure refinement for CuPP

Compound	CuPP
CCDC	2017326
Empirical formula	$C_{60}H_{84}N_2O_{10}Cu$
Formula weight	1056.83
Temperature/K	100.0
Crystal system	monoclinic
Space group	$P2_1/c$
a/Å	8.19435(8)
b/Å	17.13881(17)
c/Å	20.00751(17)
α/°	90
β/°	101.6430(9)
γ/°	90
Volume/Å^3	2752.07(5)
Z	2
ρ_{calc}g/cm^3	1.275
μ/mm^{-1}	1.040
F(000)	1134.0
Crystal size/mm^3	0.18 × 0.12× 0.07
Radiation	CuKα (λ = 1.54184)
2Θ range for data collection/°	6.86 to 152.38
Index ranges	$-10 \leq h \leq 10$, $-21 \leq k \leq 21$, $-25 \leq l \leq 25$
Reflections collected	40969
Independent reflections	5720 [R_{int} = 0.0372, R_{sigma} = 0.0166]
Data/restraints/parameters	5720/0/336
Goodness-of-fit on F^2	1.094
Final R indexes [I>=2σ (I)]	R_1 = 0.0606, wR_2 = 0.1606
Final R indexes [all data]	R_1 = 0.0617, wR_2 = 0.1613
Largest diff. peak/hole / e Å^{-3}	0.74/-0.34

Supplementary Table 10. Selected bond lengths (Å) and angels (°) of CuPP

**Selected bond lengths (Å)**						
Cu-O1	Cu-O2	O1-C1	O2-C2	O3-C4	O5-C13	O4-C11
1.9268(17)	1.9168(18)	1.303(3)	1.295(3)	1.239(3)	1.342(3)	1.274(3)

**Selected angels (°)**			
O1-Cu-O1A	180.00	O2-Cu-O2A	180.00
O1-Cu-O2	85.03(7)	O1-Cu-O2A	94.97(7)
O1A-Cu-O2A	85.03(7)	O1A-Cu-O2	94.97(7)
C1-O1-Cu	111.61(14)	C2-O2-Cu	112.23(14)

Supplementary References

1. Guo, Z. *et al.* Highly efficient and selective photocatalytic CO_2 reduction by iron and cobalt quaterpyridine complexes. *J. Am. Chem. Soc.* **138**, 9413-9416, (2016).
2. Tapodi, B. *et al.* Preparation and X-ray structure of a (catecholato)copper(II) complex with a schönberg adduct. *Inorg. Chem. Commun.* **9**, 367-370, (2006).
3. Peng, S-M. et al. Crystal and molecular structure of hydroxynaphthoquinone complex.I. The structure of bis(pyridine)bis(lawsone) copper (II) complex. Proc. Natl. Sci. Counc. B, ROC 5(2),139-144, (1981).
4. Salunke-Gawali, S., Rane, S. Y., Puranik, V. G., Guyard-Duhayon, C. & Varret, F. Three dimensional hydrogen-bonding network in a copper complex of 2-hydroxy-1,4-naphthoquinone: Structural, spectroscopic and magnetic properties. *Polyhedron* **23**, 2541-2547, (2004).
5. Gokhale, N. H. *et al.* Transition metal complexes of buparvaquone as potent new antimalarial agents: 1. Synthesis, x-ray crystal-structures, electrochemistry and antimalarial activity against plasmodium falciparum. *J. Inorg. Biochem.* **95**, 249-258, (2003).
6. Farfán, R. A. *et al.* Structural and spectroscopic properties of two new isostructural complexes of lapacholate with cobalt and copper. *Int. J. Inorg. Chem.* **2012**, 973238, (2012).
7. Hernández-Molina, R. *et al.* Complexes of Co(II), Ni(II) and Cu(II) with lapachol. *Polyhedron* **26**, 4860-4864, (2007).
8. Mejía, E. *et al.* A noble-metal-free system for photocatalytic hydrogen production from water. *Chem. Eur. J.* **19**, 15972-15978, (2013).
9. Steinlechner, C. *et al.* Selective earth-abundant system for CO_2 reduction: Comparing photo- and electrocatalytic processes. *ACS Catal.* **9**, 2091-2100, (2019).
10. Zhang, X., Cibian, M., Call, A., Yamauchi, K. & Sakai, K. Photochemical CO_2 reduction driven by water-soluble copper(I) photosensitizer with the catalysis accelerated by multi-electron chargeable cobalt porphyrin. *ACS Catal.* **9**, 11263-11273, (2019).
11. Sakaguchi, Y., Call, A., Cibian, M., Yamauchi, K. & Sakai, K. An earth-abundant system for light-driven CO_2 reduction to CO using a pyridinophane iron catalyst. *Chem. Commun.* **55**, 8552-8555, (2019).
12. Takeda, H. *et al.* Highly efficient and robust photocatalytic systems for CO_2 reduction consisting of a Cu(I) photosensitizer and Mn(I) catalysts. *J. Am. Chem. Soc.* **140**, 17241-17254, (2018).
13. Rosas-Hernández, A., Steinlechner, C., Junge, H. & Beller, M. Earth-abundant photocatalytic systems for the visible-light-driven reduction of CO_2 to CO. *Green Chem.* **19**, 2356-2360, (2017).
14. Takeda, H., Ohashi, K., Sekine, A. & Ishitani, O. Photocatalytic CO_2 reduction using Cu(I) photosensitizers with a Fe(II) catalyst. *J. Am. Chem. Soc.* **138**, 4354-4357, (2016).
15. Bonin, J., Robert, M. & Routier, M. Selective and efficient photocatalytic CO_2 reduction to CO using visible light and an iron-based homogeneous catalyst. *J. Am. Chem. Soc.* **136**, 16768-16771, (2014).
16. Rao, H., Bonin, J. & Robert, M. Non-sensitized selective photochemical reduction of CO_2 to CO under visible light with an iron molecular catalyst. *Chem. Commun.* **53**, 2830-2833, (2017).
17. Bonin, J., Chaussemier, M., Robert, M. & Routier, M. Homogeneous photocatalytic reduction of CO_2 to CO using iron(0) porphyrin catalysts: Mechanism and intrinsic limitations. *ChemCatChem* **6**, 3200-3207, (2014).
18. Rao, H., Lim, C.-H., Bonin, J., Miyake, G. M. & Robert, M. Visible-light-driven conversion of CO_2

to CH_4 with an organic sensitizer and an iron porphyrin catalyst. *J. Am. Chem. Soc.* **140**, 17830-17834, (2018).
19. Rao, H., Bonin, J. & Robert, M. Visible-light homogeneous photocatalytic conversion of CO_2 into CO in aqueous solutions with an iron catalyst. *ChemSusChem* **10**, 4447-4450, (2017).
20. Chen, L. *et al.* A molecular noble metal-free system for efficient visible light-driven reduction of CO_2 to CO. *Dalton Trans.* **48**, 9596-9602, (2019).
21. Zhang, J.-X., Hu, C.-Y., Wang, W., Wang, H. & Bian, Z.-Y. Visible light driven reduction of CO_2 catalyzed by an abundant manganese catalyst with zinc porphyrin photosensitizer. *Appl. Catal. A.* **522**, 145-151, (2016).
22. Wang, Y., Gao, X.-W., Li, J. & Chao, D. Merging an organic TADF photosensitizer and a simple terpyridine-Fe(III) complex for photocatalytic CO_2 reduction. *Chem. Commun.*, (2020).
23. Grodkowski, J. *et al.* Reduction of cobalt and iron phthalocyanines and the role of the reduced species in catalyzed photoreduction of CO_2. *J. Phys. Chem. A* **104**, 11332-11339, (2000).
24. Cometto, C. *et al.* A carbon nitride/Fe quaterpyridine catalytic system for photostimulated CO_2-to-CO conversion with visible light. *J. Am. Chem. Soc.* **140**, 7437-7440, (2018).
25. Lian, S., Kodaimati, M. S. & Weiss, E. A. Photocatalytically active superstructures of quantum dots and iron porphyrins for reduction of CO_2 to CO in water. *ACS Nano* **12**, 568-575, (2018).
26. Bi, Q.-Q. *et al.* Selective photocatalytic CO_2 reduction in water by electrostatic assembly of CdS nanocrystals with a dinuclear cobalt catalyst. *ACS Catal.* **8**, 11815-11821, (2018).
27. Kuehnel, M. F. *et al.* ZnSe quantum dots modified with a Ni(cyclam) catalyst for efficient visible-light driven CO_2 reduction in water. *Chem. Sci.* **9**, 2501-2509, (2018).
28. Kuehnel, M. F., Orchard, K. L., Dalle, K. E. & Reisner, E. Selective photocatalytic CO_2 reduction in water through anchoring of a molecular Ni catalyst on CdS nanocrystals. *J. Am. Chem. Soc.* **139**, 7217-7223, (2017).
29. Lian, S., Kodaimati, M. S., Dolzhnikov, D. S., Calzada, R. & Weiss, E. A. Powering a CO_2 reduction catalyst with visible light through multiple sub-picosecond electron transfers from a quantum dot. *J. Am. Chem. Soc.* **139**, 8931-8938, (2017).
30. Call, A. *et al.* Highly efficient and selective photocatalytic CO_2 reduction to CO in water by a cobalt porphyrin molecular catalyst. *ACS Catal.* **9**, 4867-4874, (2019).
31. Rao, H., Schmidt, L. C., Bonin, J. & Robert, M. Visible-light-driven methane formation from CO_2 with a molecular iron catalyst. *Nature* **548**, 74-77, (2017).
32. Rao, H., Bonin, J. & Robert, M. Toward visible-light photochemical CO_2-to-CH_4 conversion in aqueous solutions using sensitized molecular catalysis. *J. Phys. Chem. C* **122**, 13834-13839, (2018).
